# Functional proteins through green refining of seafood side streams

**DOI:** 10.3389/fnut.2022.974447

**Published:** 2022-08-25

**Authors:** Vazhiyil Venugopal, Abhilash Sasidharan

**Affiliations:** Department of Fish Processing Technology, Kerala University of Fisheries & Ocean Studies, Kochi, Kerala, India

**Keywords:** functional proteins, seafood discards, marine biotechnology, green processing, marine biorefinery, protein utilization

## Abstract

Scarcity of nutritive protein is a major global problem, the severity of which is bound to increase with the rising population. The situation demands finding additional sources of proteins that can be both safe as well as acceptable to the consumer. Food waste, particularly from seafood is a plausible feedstock of proteins in this respect. Fishing operations result in appreciable amounts of bycatch having poor food value. In addition, commercial processing results in 50 to 60% of seafood as discards, which consist of shell, head, fileting frames, bones, viscera, fin, skin, roe, and others. Furthermore, voluminous amounts of protein-rich effluents are released during commercial seafood processing. While meat from the bycatch can be raw material for proteinous edible products, proteins from the process discards and effluents can be recovered through biorefining employing upcoming, environmental-friendly, low-cost green processes. Microbial or enzyme treatments release proteins bound to the seafood matrices. Physico-chemical processes such as ultrasound, pulse electric field, high hydrostatic pressure, green solvent extractions and others are available to recover proteins from the by-products. Cultivation of photosynthetic microalgae in nutrient media consisting of seafood side streams generates algal cell mass, a rich source of functional proteins. A zero-waste marine bio-refinery approach can help almost total recovery of proteins and other ingredients from the seafood side streams. The recovered proteins can have high nutritive value and valuable applications as nutraceuticals and food additives.

## Introduction

Proteins are essential for healthy living. Nutritive proteins as well as bioactive peptides play a vital role in the growth and development of living systems. Their functional roles nuclide cell and extracellular structures, enzyme-catalyzed reactions, gene expression, hormone-mediated actions, muscle contraction, osmotic regulation, protection against oxidative stress, immunological protections, metabolic regulation, storage and transport of oxygen (1). Adequate intake of dietary proteins can alleviate problems associated with muscle loss in the elderly and retarded growth among children. The availability of nutritive proteins for global population, however, is far from adequate. The Food and Agriculture Organization (FAO) has observed that approximately one billion people in the world do not receive sufficient amounts of dietary proteins (2). Malnutrition is likely to reach serious proportions as the global population increases to about 9.8 billion by 2050 (3). Plant and animal-based foods supply about 65% and 35 of proteins, respectively. These encompass meat from land animals, eggs, poultry, seafood, dairy products, beans, peas, soy products, nuts, seeds, and others. Plant proteins, however, are “incomplete” in that they can be deficient in one or more of nutritionally indispensable amino acids, known as essential amino acids (EAA), which include lysine, methionine, threonine, tryptophan, isoleucine, leucine, phenylalanine, and valine. Therefore, the average diet may require proteins from multiple plant sources so as to compensate nutritional deficiencies (4). Increased use of meat of land animals as protein source is known to generate greenhouse gases besides requiring large volumes of water and land for farming. Further, beef cattle have a poor feed bioconversion rate for the output of edible proteins. It is therefore important that the demand for proteins needs to be met with sustainable approaches. Efforts in this regard include promoting biodiversity within the food production systems, development of alternate proteins via regenerative techniques as well as reclaiming nutritive and sustainable proteins from waste streams. The recovered proteins should meet the nutritional requirements, environmental safety standards and also consumer acceptance (2, 3). Food production, postharvest handling and storage result in voluminous discards. These discards, which are rich in proteins, can be sources of proteins and other value added products (5). Attempts have been made to recover proteins from seafood discards. The conventional chemical processes employ harsh chemicals such as mineral acids and alkali, which cause adverse effects on the environment. Besides, the processes are costly, time consuming, and require large volumes of water for neutralization and washing off of acids and alkali. Therefore, novel eco-friendly technologies are essential for safe and sustainable recovery of proteins and other compounds from seafood discards. This article discusses potential benefits of green processing along with algal biotechnology and marine biorefinery for zero-waste bioconversion of seafood side streams, with particular reference to the recovery of proteins. The article also briefly addresses functional and bioactive properties of recovered proteins, and scope for their uses as healthcare supplements, nutraceuticals, food additives and many others, It is also pointed out that the approach can have economic advantages as it can address global protein scarcity, besides supporting a circular bioeconomy.

## Seafood production

Fish is critically important to food security and good nutrition. The annual global seafood production has been around 170 metric tons (MT) in the last several years. In 2016, the global seafood production was 170.9 MT, which included 90.9 MT capture, consisting of 79.3 and 11.6 MT of marine and inland fish, respectively. Capture fisheries resulted in 7.14 MT crude proteins against 6.82 MT of proteins derived from aquaculture (6). Aquaculture, with a production of 80 MT in the 2016, which included finfish, mollusks, and crustaceans at 54.1, 17.1, and 7.9 MT, respectively, has grown at an annual rate of 5.8% during the period 2000–2016. Global fish production is projected to reach 200 MT by 2029, increasing by 25 MT (or 14%) from the base period (average of 2017–19). It is projected that utilization of fishery products for human consumption will reach 180 Mt by the year 2029 from the 2018 figure of 129 MT. The consumption of seafood over the next decade is expected to keep rising at a faster rate than meat consumption; about 58% of this amount coming from aquaculture (7). Based on status quo consumption, aquaculture production alone would need to be 129 MT by 2050 to meet the increasing demand for proteins (8).

### Seafood side streams

It is well-known that all the seafood catch is not completely used as food. In the year 2018, about 22 MT or 23.9% of the total capture fishery were not used for human food (9). The average annual discards in global marine capture fisheries were estimated to be 10.5 MT. An estimated 9.4% of the annual average catch during the period 2010–2014 was discarded (10). In addition to the bycatch, the seafood industry, which processes about 80% of the harvest into diverse products, leaves 30 to 70% of the raw material as process discards (11). The crustacean discards are composed of cephalothorax, carapace, shell and tail, which amount as high as 6 to 8 MT annually, worldwide (12). In India the shrimp processing industry yearly generates about 100,000 tons of shell waste (13). The shrimp waste contains about 70% head and 30% shell of the crustacean, the shell portion consisting of 20 to 40% proteins, besides 15 to 40% chitin (14). Lobster processing wastes can be 50 to 70% of the whole shellfish, with release of more than 32 MT discards (15–17). Discards from finfish constitute 25 to 50% of the raw material, and comprises of entrails, heads, skeletal frames, skin, scales and viscera. Canning of tuna results in as much as 70% solid wastes consisting of muscle, dark flesh, head, bone, and skin. Fileting of large fish such as hake, seer, and others generate frames that carry meat portions up to 60% of weight of the fish. Pre-processing of freshwater fish such as trout, carp, pike-perch, pike and bream into various products such as gutted, headed, and free or skin on filets generates 40 to 60% of the fish as waste. Freshwater fish such as perch, bream, pike-perch and carp are rich in thick scales. Approximately 49,000 MT of fish scales have been reported to be produced annually (18, 19). In addition, seafood processing is accompanied by the generation of voluminous amounts of effluents. The industry including aquaculture routinely uses voluminous amounts of fresh water and discharges large volumes of post-process wastewater as effluents. Processes such as marination and surimi production also generate large volumes of waste water (20, 21).

## Seafood side streams as source of proteins

Fishery products are an important part of healthy diet. The contents of nutritive proteins and also other nutrients give this food group a healthy image among nutritionists as well as consumers. Their rich nutritive proteins are vital in influencing health of the consumer (22, 23). Seafood muscle contains proteins ranging from 18 to 23% depending on nature of the fishery product (finfish, shellfish or cephalopods), and their habitats (marine water, freshwater, or brackish water). They may be pelagic (fast-moving species on surface waters such as tuna, herring, mackerel, sprat, anchovy, and sardine) or demersal (slow-moving species in deep waters such as cod, deepwater shrimp, etc.) nature. Apart from their muscle, proteins are found in appreciable quantities in bones, head, viscera, liver, kidney, eggs, and skin. For example, in oysters, proteins are mainly distributed in the soft body including visceral mass, mantle, gill, and adductor muscle. The proteins include myofibrillar or structural proteins consisting of myosin, actin, actomyosin, and others that are soluble in aqueous salt solutions of ≥0.3 ionic strength, sarcoplasmic proteins including myoglobin, hemoglobin, globulins, albumins, and various enzymes (soluble in water or low ionic strength solutions), and connective tissue proteins including collagen, elastin, and gelatin. Myofibrillar proteins constitute 65 to 75% (w/w) of the total proteins of fish and shellfish muscle. Shellfish, in general, contains slightly more proteins than finfish. A novel protein, paramyosin (molecular weight 200 to 250 kDa), is also found at varying levels in invertebrate myofibrils, but not in vertebrate myofibrils. Myosin, the main structural protein, is a long rod with two globular heads at one end, and tail portion, which has a total length of 155–160 nm with a molecular weight of ~500 kDa. It consists of two large (2,00,000 Da each) and 4 small (20,000 Da each) subunits. The structural proteins have good functional properties (23, 24).

The current global per capita consumption of seafood is 20.1 kg that provides about 20% of total average intake of animal proteins (9). Boyd et al. (8) recently determined availability of protein for human consumption from different animal food sources. Based on 2018–2019 database of production, they calculated that meat of land animals, milk, and eggs offered 76,966 kilo ton (Kt) crude protein. Land animals provided 37,391 Kt of proteins, equivalent to 41.1% of total proteins, followed by milk (30, 889 Kt; 34.4% of total proteins), and eggs (8,686 Kt; 9.6%) proteins. An amount of 13,950 Kt of proteins was contributed by aquatic animals in 2018, which was 15.3% of total available proteins. The aquatic products included capture fisheries, which provided 7,135 Kt of crude proteins while aquaculture supplied 6,815 Kt proteins. The data also showed that aquaculture does not lag behind capture fisheries in protein supply (8).

The bycatch, process discards and also effluents, collectively designated “seafood side streams” in this article, represent good source of proteins, besides many other nutrients (23, 25–29). The challenges of sustainable seafood processing are linked to making use of these seafood side streams for food, reduction of environmental pollution, and conservation of water. Sound management of the side streams can therefore lead to sustainable fisheries and healthy ecosystems, and long-term food security (10, 30). At the 32nd Session of the Committee on Fisheries, held in July 2016, focus was placed on the 2030 Agenda for Sustainable Development. The Sustainable Development Goal (SDG) #14 is one of the 17 Sustainable Development Goals established by the United Nations in 2015. The SDG calls for conservation and sustainable use of the oceans, seas and marine resources for sustainable development (31). The SDG #12.3 aims at halving global food waste including waste from marine sources at the retail level and by reducing food losses including post-harvest losses along the production and supply chains by the year 2030 (32). There is immense potential for the use of seafood side streams as low cost, renewable feedstock for functional proteins, besides other therapeutic and industrially important ingredients.

### Seafood bycatch as source of protein-rich edible products

**Figure 1:**
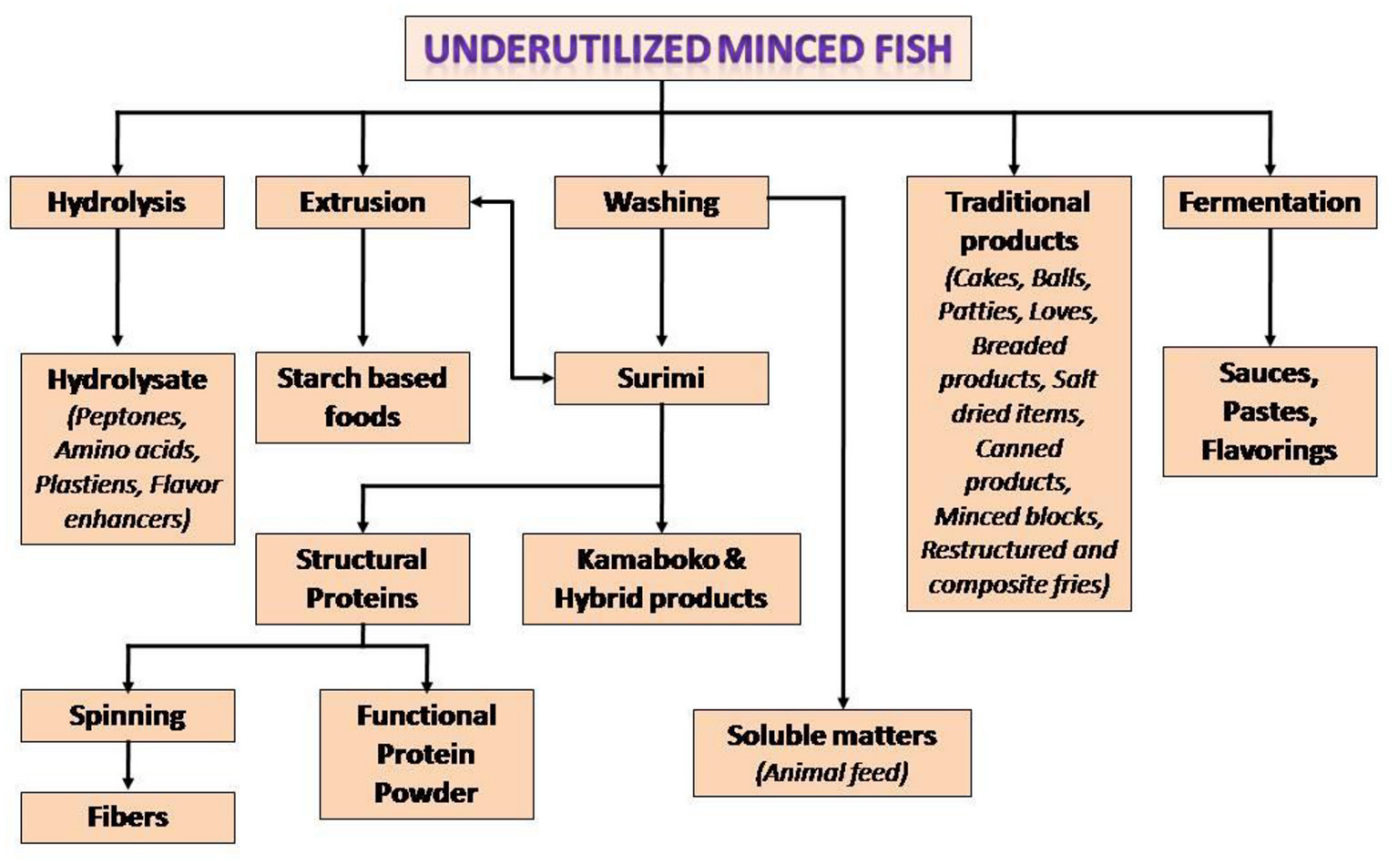
Potentials for various protein-rich products from bycatch meat. Source, Venugopal and Shahidi (33) with permission.

The concerns with respect to landing of large volumes of seafood as bycatch have encouraged a number of countries in the Organization for Economic Cooperation and Development (OECD) formulate practices to reduce such landings (10). The bycatch can be used for the development of protein-rich edible products. The process involves initial recovery of meat from these fish by mechanical deboning (The technique can also be employed to recover meat from fish frames from fileting operations). The recovered mince can be used to prepare food items such as surimi-based restructured products, sausages, noodles, fermented, extruded and coated products, protein concentrates, hydrolysates, and many others (33). For making surimi (washed concentrate of myofibrillar proteins), the fish mince is repeatedly washed with chilled water to remove soluble components such as pigments, enzymes and also lipids adhering to the fish meat. The surimi can be kept frozen in presence of cryoprotectants to retain the protein functionality. Surimi forms gel under warm temperature conditions in presence of small concentrations of salt. Surimi gel can be used as raw material for restructured imitation products such as shrimp, crab, etc. Conventionally Alaska Pollock is used for surimi preparation because of favorable functional properties of its surimi. The decline in the landing of Alaska Pollock has led to evaluation of other species for surimi production. Some of the fish successfully examined include croaker, barracuda, threadfin bream, lizardfish, cutlass fish, stripped mullet, leather jacket, sea bream, puffer and red big eye and others (21, 23). Surimi can be dehydrated to protein powder in presence of cryoprotectants such as sucrose and polyols in order to prevent denaturation of the proteins during drying and storage (34). The bycatch can be raw material for consumer acceptable surimi-based restructured products and also surimi powder. Other likely products from these fish include filets, paste, burgers; and extrusion cooked, smoked and dehydrated items (35, 36). Figure 1 shows potentials for various protein-rich products from the meat of bycatch fish.

### Protein contents of seafood discards

Seafood discards, on dry weight basis, have average 60% proteins, in addition to 19% fat and 21% ash (37). Shrimp head waste can contain up to 65% proteins along with 20% ash and 18% chitin (18). Liu et al. (38) reported meat contents of muscle, head, shell and tail of five species of shrimp. The shellfish yielded meat ranging from 37 to 56%, while proteins in their byproducts ranged between 44 to 63%. Lobster liver contains up to 41% protein on a dry basis, while lobster shells carried about 25% proteins. The Australian rock lobster heads contain about 44% protein. Lobster meat has 39 to 41% EAA (15). Collagen is the principal fibrous and structural protein of extracellular matrix of animals, which contributes to physiological functions of tissues in cartilage, skin, bones, and tendons. Finfish has significant levels of collagen in their skins, scales and fins. Amino acid composition determines collagen types. As high as 29 distinct types of collagens has been recognized. Fish gelatin could be extracted from denatured collagen, usually by hot water treatment (26, 39).

### Proteins contents of process effluents

The effluents released from seafood processing plants contain significant quantity of organic matter. This is in the form of total suspended solids (TSS), fats, oils, and grease (FOG), excess nutrients (nitrogen and phosphorus), and proteins. The proteins consist of myofibrillar proteins, collagen, gelatin, enzymes, peptides and amino acids, which remain in soluble, colloidal or particulate forms. The water from processing of fish species including Pollock, cod and salmon had ~6% proteins besides minerals and oil. Discharge of these effluents without treatment is highly hazardous to the environment (20). Table 1 shows protein contents of various seafood side streams.

**Table 1:** Protein contents of seafood side streams.

**Fishery source**	**Protein contents (g%), wet weight**	**Derived protein products**
Finfish heads	11.9–12.9	Proteins, protein hydrolysates, biopeptides
Finfish frames	11.5–17.5	Collagen, gelatin, protein hydrolysates, biopeptides
Finfish Skins and fins	24.8–27.0	Collagen, gelatin, protein hydrolysates, biopeptides
Finfish Viscera (livers, roes, and milts)	12.9–14.8	Enzymes, protein hydrolysates, peptides, biopeptides
Crustacean shells	29.0–40.0	Proteins, caroteno-proteins
Crustacean heads	43.5–54.4	Shell and meat proteins
Crustacean Viscera	41–43	Enzymes, protein hydrolysates, peptides, biopeptides
Lobster head	43.5	Proteins
Molluscs (Oyster, Mussel, Clam, Scallop) body parts, organs	58.7	Enzyme, protein hydrolysate, biopeptide, food flavor
Dry seafood waste	60 (dry wt. basis)	Miscellaneous proteins

Adapted from Islam et al. (37).

## Green processes for the recovery of proteins

Green processes are upcoming options to replace conventional technologies for the extraction of ingredients from food wastes because of their ease of handling and environmental safety. Borefining is an innovative and efficient approach for the sustainable processing of biomass into a spectrum of marketable bio-products and bioenergy/biofuels (40). Green chemistry based biorefining is safe, efficacious, ecologically friendly, and also does not adversely affect intrinsic characteristics of the extracted compounds. Considering the yield and quality in terms of energy and cost green technologies can show promising results for recovering bioactive compounds than conventional methods (16, 27, 41–43). The green processes for resource recovery include microbial fermentation, enzymatic processes, methanogenesis, photosynthesis, oleaginous processes, and others. Novel non-thermal physico-chemical processes encompass ingredient extractions assisted by ultrasound, high hydrostatic pressure (HHP), microwave, pulsed electric field (PEF), dense phase carbon dioxide (DPCD), subcritical and supercritical fluids, membrane filtrations and enzyme treatments (42, 44–46). Green solvents can replace petroleum based solvents to recover the isolated compounds. These solvents include water, supercritical fluids, ionic liquids, liquid polymers and their varied combinations. Acetone, ethanol, methanol, 2-propanol, ethyl acetate, isopropyl acetate, methyl ethyl ketone, 1-butanol have been included in this category. They are characterized by low toxicity, minimum environmental impact, convenient accessibility and possibility of reuse as well as high extraction efficiency (12, 47). Membrane-based separation processes have emerged as novel tools to efficiently separate proteins, and other organic matter from extracts. The membrane processes include ultrafiltration (UF), nanofiltration (NF), microfiltration (MF), reverse osmosis (RO), and forward osmosis (FO). Membranes may be made of nylon, polyether sulfone, polyvinylidene difluoride, mixed cellulose ester, cellulose acetate, polypropylene and other materials. These processes are used depending on mol. wt. requirements of the desired final products. Nanotechnology can have a positive impact in the development of membranes. Membrane processes may involve problems such as membrane fouling and may need cleaning of fouled membranes (48).

Green processes are promising alternatives for the extractions of proteins and other biomolecules from seafood side streams (42, 45, 46, 49–51). The major green processes with particular reference to recovery of seafood proteins will be discussed.

### Bioconversions by microbial fermentation

The two major green processes for bioconversion employ both microbial fermentation and treatment by enzymes (52). Fermentation results in the production of hydrolytic enzymes such as proteases, chitinases and lipases, and others depending on the microorganism. Microbial fermentation using fish waste as a source of carbon and nitrogen is considered a low-cost, safe, and sustainable technique to obtain a wide range of valuable compounds from food wastes. Bioconversion of feedstock resources makes use of aerobic, anaerobic, or facultative bacteria, fungi, mycelium, or microalgae. Lactic acid bacteria (LAB) such as *Streptococcus thermophilus, Lactobacillus acidophilus* and *L. bulgaricus*, which produce lactic acid from sugars, are popular organisms for fermentation. During LAB fermentation, the acid-induced low pH enhances activity of acid proteases, which release proteins that are bound to lipids, carbohydrates, minerals and carotenoids in the waste. The acid also reacts with calcium carbonate present in side streams, particularly shellfish discards, to form calcium lactate; the low pH also controls of proliferation of contaminant microorganisms. The proteases assist in the deproteination and demineralization of the food waste. The fermentation efficiency can be improved by selecting specified operating conditions such as solid or liquid state, submerged, anaerobic, continuous or fed batch, depending upon the nature of the microorganism used (53). Over the past century, the role of fermentation has expanded to a broader range of applications, spanning industrial chemistry, biomaterials, advanced food ingredients, therapeutics, medicine, and fuels. These developments make use of “biomass fermentation” instead of traditional fermentation. Fermentation is the backbone in the newly developing alternative protein industry (54). Developments in fermented fish products are of crucial importance not only for the food industry but also for human health (55).

Microbe-assisted bioconversion is ideal for bio-refining of seafood processing wastes such as viscera, shells, and heads and to develop quality protein hydrolysates, bioactive peptides and other compounds such as chitin and oil (56). These processes can result up to 80 to 90% of demineralization and deproteinization of crustacean shell waste. Fermentation of shrimp shell waste by symbiotic LAB for 168 h decreased the substrate pH to about 4.2 and promoted removal of 98% protein, 91% calcium and 32% carotenoids. About 88% of peptides in the hydrolysate had mol.wts, ranging between 1,000 and 10,000 Da, and the remaining below 10,000 Da (57). Chemical pretreatment of collagen raw materials is time-consuming and environmentally hazardous. Therefore, Song et al. (58) used fermentation to extract acid-soluble collagen (ASC) and pepsin-soluble collagen (PSC) from Nile tilapia. The yields were comparable to conventional chemical extractions. The extract contained type I collagen, which retained its triple helical structure well. The collagens had denaturation temperature between 36.0 to 37°C, and high solubility under acidic conditions. The proteolytic treatment of minerals-rich grass fish bones followed by microbial fermentation gave soluble calcium salts of lactate and acetate, and also calcium containing peptides, which can be used as calcium supplements (59). Jung et al. (60) employed *L. paracasei* followed by a protease producing bacterium to remove 70% proteins, 94% CaCO_3_ and chitin from crab shell. A novel consortium of LAB was used for the fermentation of shrimp head waste, which allowed maximum recovery of protein-rich liquor. The liquor when added to commercial powdered fish feed at 30% (*w/w*) helped Nile tilapia larvae achieve maximum survival, weight and length gains, specific growth rate and biomass formation (61). Fermentation of prawn shell waste by *Bacillus megatarium* in presence of 0.1% glucose resulted in 73% demineralization and 73.28% deproteination, resulting in 40% recovery of chitin along with protein hydrolysate (49).

### Enzymatic bioconversions

Enzymatic treatments have significant benefits in waste management. The usage of endogenous enzymes (isolated from fish or shellfish) in the seafood industry is reported to reduce environmental pollution, valorization of seafood waste, replacement of conventional thermal processes and development of novel products (62, 63). Hydrolases are the popular enzymes in bio-processing, which include proteases, carbohydrases and lipases. Specific, energy-efficient enzymatic techniques using proteases and also other enzymes are novel techniques for seafood processing. Commercial enzymes include alcalase, neutrase and flavorzyme. Enzymatic hydrolysis of proteins from aquatic byproducts and also livestock, poultry, and plants offer novel applications in foods, pet feed, pharmaceutical, and other industries (64). Rodriguez et al. (65) isolated proteinases from fishery wastes having acid and alkaline activities. The enzymes could digest feed supplements to increase digestion efficiency in tilapia fingerlings and juveniles, but did not affect the activity and integrity of fish digestive enzymes.

Recent developments for large scale enzymatic extractions of biomolecules include single or multiple extractions, coupling enzymatic processes with other technologies such as ultrasound, microwave, high pressure and supercritical CO_2_ extractions. Immobilization of enzymes on magnetic nanoparticles can enhance the operational performance by the possibility of multiple uses. These processes are industrially and economically feasible (66). Treatment of prawn shell waste with immobilized chitinase resulted in high level of deproteinization. The immobilized enzyme had superior activity and stability, suggesting potentials for its repeated use. The proteins could be recovered by flocculation followed by membrane filtration (13). Some of the aspects that need to be considered in enzymatic extraction include pre-treatment requirements, compatibility of components in the food matrix and interactions of components among themselves, nutritional and product safety (67, 68). Table 2 present some examples of fermentative and enzymatic extractions of proteins from seafood discards.

**Table 2:** Examples of fermentative and enzymatic extractions of proteins from seafood side streams.

**Seafood discards**	**Enzyme used**	**Protein types**	**References**
Fishfin, scales, head	Collagenase, trypsin	Collagen	(63)
Fish waste	Trypsin, alcalase, pepsin	Protein hydrolyzate	(63)
Shell waste	Microbial action Chitinase, protease		(69)
Shell waste	Fermentation	Protein	(70)
Shrimp shell	Fermentation	Protein feed	(61)
Shrimp and crab shell, squid pen	*Pseudomonas aeruginosa* protease	Proteins and also chitin	(71)
Shrimp waste	Fermentation by lactic acid bacteria	Proteins and also chitin and astaxanathin	(72)
Crayfish waste	Simultaneous protease, fermentation	Proteins and also chitin	(73)
Aquaculture solid waste	Heterotrophic and nitrifying bacteria.	Liquid fertilizer	(74)
Lobster waste	Papain hydrolysis	Proteins and also astaxanthin	(70)
Grass fish bone	Proteolysis followed by fermentation	Calcium supplement	(59)

### Algae-assisted bioconversion

Photosynthetic organisms represent a valuable tool for waste valorization. Algal bio-technology makes use of microscopic, unicellular and photosynthetic microalgae for the extractions of ingredients present in seafood and other food discards and also fishery effluents. The technology involves growing photosynthetic microalgae in media employing seafood waste as nutrient source. Microalgae such as *Haematococcus pluvialis* and *Arthrospira* spp. (belonging to Cyanobacteria), rapidly multiply in nutrient media converting CO_2_ and nutrients into biomass. Their growth is parallel with fixation of atmospheric CO_2_ at a rate that can be as high as 1.83 kg CO_2_/kg biomass; supplementation of CO_2_ enhances biomass formation. Carbon capture by microalgae is both eco-friendly and economical. These organisms show several advantages such as low-cost production and the ability to grow rapidly using freshwater, seawater and wastewaters. Depending on the organism, their optimal growth can be indoor, under photoautotrophic, heterotrophic, or mixotrophic conditions, or in closed photo-bioreactors. Photobioreactors facilitate in maximization of solar energy capture and conversion, using sunlight, atmospheric CO_2_ and water, photosynthetic organisms belonging to Chlorophyta (green algae), Chrysophyta (golden-brown algae), and Cyanophyta (blue-green algae) represent efficient and economic platforms, which can make use of food industry wastes as nutrient media. The cultivated algal biomass, generally termed as single cell protein (SCP), contains 40 to 50% proteins on dry weight basis in addition to high-value compounds including omega-3-fatty acids, pigments, amino acids, and others. Selecting suitable algal species, nutrients and culture conditions, it is possible to optimize bioconversion and production of SCP. Field experiments have shown an approximate cost of USD 1.1 per kg SCP (75). The process also mitigates environmental hazards. The algal technology has been discussed by several authors (76–79).

### Physico-chemical processes

A number of non-thermal physic-chemical methods have been reported to be advantageous for the recovery of proteins and other ingredients from seafood side streams. Ali et al. (44) discussed advantages of non-thermal processing methods including PEF, DPCD, HHP, membrane technology, ultrasound-assisted extraction (UAE), and enzyme-assisted methods for recovery of bioactive compounds from marine by-products. UAE and SFE are emerging technologies to valorize seafood and their by-products (51). Bruno et al. (41), citing specific examples, pointed out beneficial effects of these green processes in valorization of seafood discards. Extraction of proteins from the viscera of abalone was enhanced by PEF treatment. Ultrasound assisted extraction significantly increased the yields of collagen and gelatin, as compared with conventional methods. UAE-extracted gelatin from fish scales had higher gelling and melting points, gel strength, apparent viscosity and emulsifying properties than conventionally extracted gelatin. Ultrasonication, microwave and supercritical fluid extraction were found to be promising techniques for large scale recovery of proteins from lobster discards. An integrated process of ultrasonic intensification for 5 min at a pH of 13.0 in presence of 250 mg per L chitosan recovered up to 90% proteins from lobster heads (15). High-intensity ultrasound (HIU) was combined with an alkaline pH-shift process to extract protein from tilapia (80). Ultrasound can also assist the extraction of collagen from sea bass skin (81). Supercritical fluid extraction was used for the extraction of proteins from shrimp processing waste at a yield of about 22% (82). HHP induces unfolding and/or denaturation of proteins enhancing their enzymatic hydrolysis (83). Steam assisted extraction is another promising method to recover proteins from native fish bone materials. Hydrothermal pretreatments at 159°C for 2 min, followed by treatment at 121°C for a period of 70 min optimally extracted the proteins (84). Functionally active proteins from mackerel viscera were extracted using supercritical carbon dioxide at temperatures ranging from 35 to 45°C, and at a pressure of 25 MPa (85). Green extraction is recommended for the recovery of bioactive protein hydrolysates and also enzymes from crab discards (86). Membrane processes have been successfully used for the recovery of proteins from effluents without loss of their functionality (20).Natural deep eutectic solvents (NADESs) are sustainable, non-toxic and biodegradable solvents, which are composed of primary natural metabolites. These solvents can replace ion of harsh chemicals, which are detrimental to the environment. A green and efficient approach based on choline chloride-malic acid, a NADES, extracted most of the proteins and minerals from shrimp shells with the assistance of microwave irradiation (87). Some of the other deep eutectic solvents are mixtures of betaine hydrochloride -urea, choline chloride-urea, ChCl-ethylene glycol, and ChCl-glycerol (88).

#### The pH shift process

A well-studied green process is isoelectric solubilization precipitation (ISP). The process involves homogenization of fish discards or bycatch meat portions with either dilute acid (pH 2.5 to 3.5) or alkali (pH 10.8 to 11.5). The treatment dissolves sarcoplasmic and myofibrillar proteins, while insoluble impurities such as bone, skin, oil and membranes are removed. Up to 90% of the dissolved proteins are precipitated by raising the pH of the solution to their iso-electric pH of pH 5.2 to 6.0, which are then concentrated by centrifugation or filtration. ISP has been used to recover proteins from various species of finfish and shellfish and their discards, bycatch, and also from process effluents (89). A few examples are cited. Channel catfish muscle was subjected to extraction and precipitation techniques using acid (pH 2.5) or alkaline (pH 11.0). Solubility of the fish proteins was found to be highest at pH 2.5 and 11, and at these pH levels. Viscosity was found to be low enough to cause separation of proteins from insoluble materials by centrifugation. Both the acid and alkali-aided processes led to higher protein recovery compared with proteins extracted during surimi processing. Almost all the proteins present in surimi processing water were precipitated by pH shift process followed by heat treatment at 60°C (90). Proteins have also been recovered from tilapia frames using the ISP process (91). Mechanical deboning and pH-shift processing on protein recovery from salmon, herring and cod backbones have been compared. Mechanical separation led to higher protein recovery compared with the pH-shift process. Combination of both the techniques recovered maximum proteins from herring followed by salmon and least from cod. The pH-shift process up-concentrated protein from herring and salmon backbones more efficiently than mechanical separation (92).

#### Flocculation

Recovery of proteins from seafood process effluents including surimi wash water and fishmeal stickwater can be difficult due to their relatively low concentrations. These proteins, which are present in suspended or dissolved state, can be flocculated and precipitated by food grade polysaccharides such as carrageenan, alginate or carboxy methylcellulose. The recovery rates for carrageenan, guar, chitosan, and alginate were 70, 49, 53, and 55%, respectively. The precipitated proteins are then subjected to concentration by filtration including membrane filtration, sedimentation and/or centrifugation (93, 94).

#### Membrane processes

**Figure 2:**
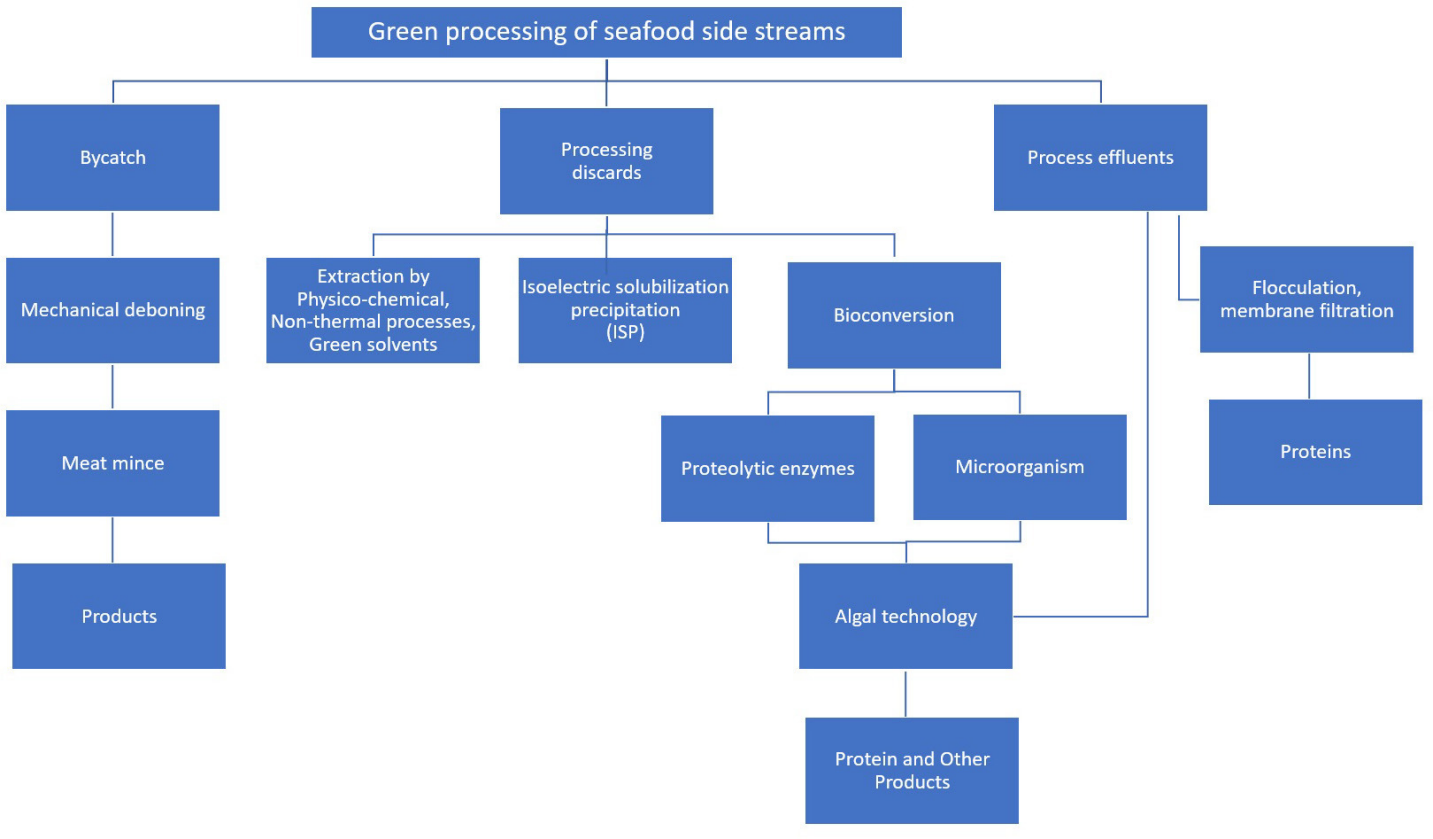
Green processes for extraction of proteins from seafood side streams.

Membrane-based separation processes have good prospects to separate proteins from seafood process effluents, protein hydrolyzates and wastewater streams at affordable cost with minimum energy requirements (20). Integration of a bioreactor with membranes, known as “membrane bioreactor” is highly useful for the separation of peptides from protein hydrolyzates (95). Ultrafiltration has been applied widely in food processing industry due to its advantages over conventional separation process. These include possibility for gentle handling of the product, high selectivity, and lower energy consumption .UF could remove up to 96% of proteins from shrimp shell extract after incubating it in dilute alkali for 2 h at 45°C, at a solid to solvent ratio of 1:2 (w/v) (96). Tonon et al. (97) coupled UF and enzymatic hydrolysis to prepare hydrolyzate of shrimp wastewater proteins. Membrane filtration concentrated proteins present in wastewater of cooked snow crab. The concentrate having 59% proteins and desirable flavor can be used as a flavor additive in the food industry (98). The UF has also been successful in recovering proteins from surimi wash water (99) and pre-salting brine from the marination of herring (100, 101). UF membrane attached bioreactors having appropriate molecular weight cutoffs were effectively used to separate peptides from fish protein hydrolyzates (102). Protein solutions obtained by different processes may be concentrated by drying by using suitable technologies such as falling film evaporators, rising film evaporators, spray drying, roller drum drying, among others (103). Figure 2 summarizes major green processes for valorization of seafood side streams.

### Bio-refinery for seafood side streams

Bioconversion methods, discussed above, combined with a bio-refinery strategy offer potentials for the isolation of multiple products from food waste, in a sustainable, environmentally-friendly and cost-effective manner (104). Analogous to the petroleum refinery, the bio-refinery supports sustainable green pathways to produce marketable bio-based products. It visualizes a closed loop approach wherein waste is valorized through a cascade of green processes addressing circular economy. The circular economy concept envisages a system in which the final disposal of waste is minimized by promoting their reuse and valorization (105). In a waste bio-refinery, each constituent of the feedstock resource is extracted and functionalized in order to produce food and non-food fractions including intermediate agro-industrial products. Biotechnology is important with biotransformation by microbial fermentation and enzymatic action taking pivotal roles in waste valorization (106). Kamm and Kamm (107) classified bio-refinery into three types: (1) phase I, which uses only one feedstock material, has fixed processing capability, and produces a single primary product; (2) phase II, uses only one feedstock, but produces multiple products; and (3) phase III, produces different types of raw materials, processing technologies, and produce different types of products. Kamm et al. (108) also proposed another classification based only on the type of feedstock used: (i) lignocellulosic bio-refinery; (ii) whole crop bio-refinery; (iii) green bio-refinery; and, (iv), the organic waste bio-refinery. The bio-refinery approach is receiving attention for sustainable and holistic utilization of waste (105, 109).

#### Marine bio-refinery

A marine bio-refinery envisages conversion of oceanic resources into multiple value added products such as proteins, lipids, minerals, pigments, chitin and others for use as neutraceuticals, food, animal feed, organic fertilizer, biofuels, and others (49, 110). It must be emphasized that the recovery of a single product from a bio-refinery including marine biorefinery is unprofitable and generates undesirable waste. The marine bio-refinery therefore aims at integration and optimization of preprocessing, cultivation, and extraction for efficient, sustainable, and profitable utilization of oceanic products on a zero-waste approach. This approach allows ocean-based industries convert low-value biomass into commercially relevant by-products. The development and implementation of a successful marine bio-refinery can meet the goals of a “greener” socioeconomic development, also termed “blue economy”; a term is used as a synonym for “sustainable ocean-based economy” (111). The development and implementation of a marine biorefinery can be fundamental to consolidate a “greener” socioeconomic oceanic development (112).

A number of marine bio-refinery-based approaches have been examined to utilize seafood side chains for the recovery of proteins (and also other valuable ingredients). Successful operation of a biorefinery can be environmental friendly, as determined by life cycle assessment (LCA) (12).

#### Crustacean waste bio-refinery

**Figure 3:**
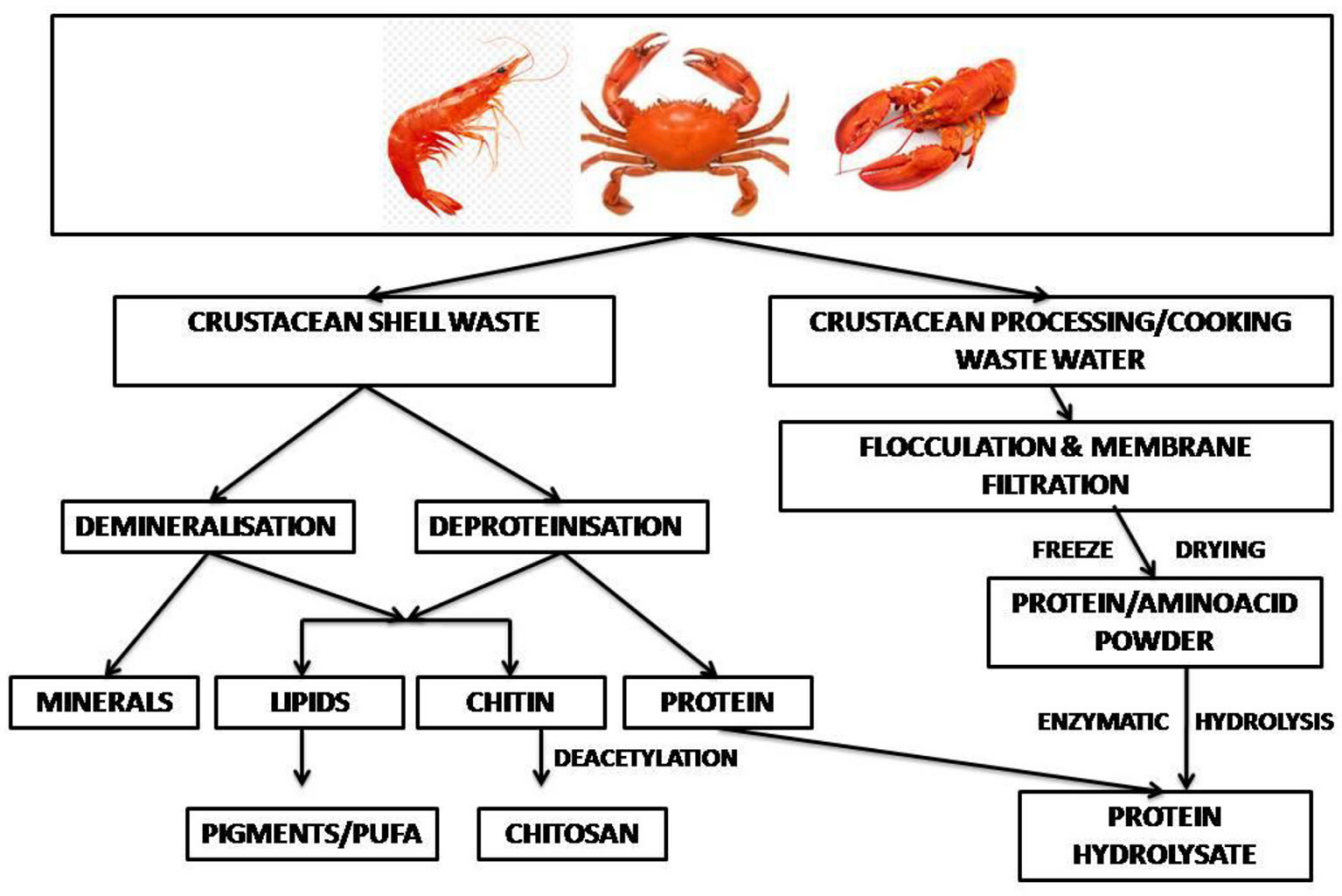
Schematic diagram for **c**rustacean waste bio-refinery.

Valorization of wastes based on the circular economy approach is the key to cleaner and more efficient production in the shrimp industry (113). In a shell refinery, the crustacean waste is subjected to sequential treatments to recover chitin, proteins, lipids, carotenoids, calcium carbonate and chitin monomers (82, 114). In an enzyme-based bio-refinement process recombinant aspartic proteases were used for protein hydrolysis, together with recombinant chitinase for chitin hydrolysis, and ethyl acetate, a green solvent, for extraction of astaxanthin pigment. The process offered zero-waste shell utilization with recovery of about 92% protein and 89% chitin (115). Cahu et al. (116) reported an integrated process to recover protein along with chitin, carotenoids and sulfated- and amino-polysaccharides from shrimp heads. About 120 g of protein hydrolysate was recovered per kg wet processing waste. A two-stage solid state culture by *L. brevis* followed by *Rhizopus oligosporus* resulted in 96% deproteinization of crustacean shell waste. The released protein hydrolysates had mol. wts in the range of 11,000 and 25,000 Da, and had antioxidant activities (117). Yang et al. (118) reported an integrated eco-friendly process for the recovery of multiple components from crustacean shell waste. Hot water was used for deproteinization and carbonic acid for demineralization, which yielded as high as 90% deproteinization and demineralization within a few hours. Equipments required by the refinery included crusher, mixer, deproteinization and demineralization reactors, mineral separator drum, vacuum drum filter, protein separator drum, multi-stage solid washer, air-dryer drum, and others (118). Lactic acid fermentation followed by green processes can sequentially or simultaneously extract hydrolyzed protein, astaxanthin and chitin from marine wastes (70). The application of a sequential enzymatic-acid-alkaline treatment for chitin extraction from shrimp cephalothorax at pilot plant scale released proteins (119). Vicente et al. (12) used harmless solvents, namely water, the protonating acetic acid under mild functional conditions and buffers, conjugated with solid–liquid extraction, centrifugation, and membrane filtration to develop a sustainable, environmentally friendly shell waste bio-refinery. The shrimp biorefinery topology showed attractive findings from a technical, economic and environmental point of view (113). A general perspective on the techno-economic feasibility and the environmental footprint of the biorefinery processes for fishery waste has been discussed (27). A bio-refinery developed within an EU-funded project, combines microbial demineralization of novel bio-based polymers (120). A schematic diagram of shellfish waste bio-refinery is depicted in Figure 3.

#### Finfish waste biorefinery

**Figure 4:**
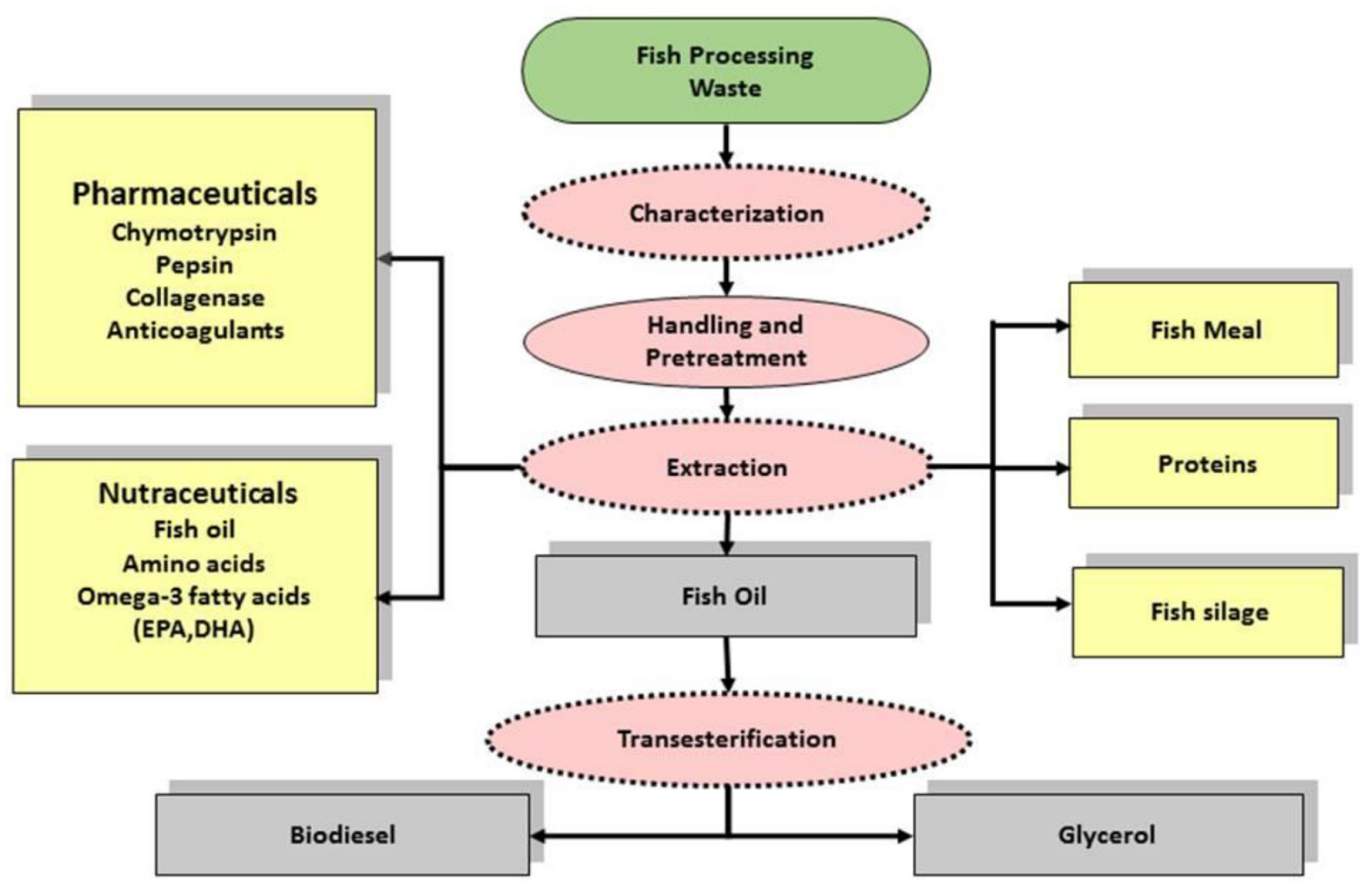
Schematic diagram for finfish waste bio-refinery. Source: Vegneshwaran and Dave (125), with permission.

Finfish processing waste can be a promising renewable biomass resource for valorization by bio-refineries. Vázquez et al. (121) employing a combination of enzymatic, fermentation and chemical processes isolated gelatin, bioactive peptides rich fish protein hydrolysate (FPH) and other compounds from skin and heads of fish including megrim, hake, boarfish, grenadier, and Atlantic horse mackerel. All the FPHs yielded 13% of hydrolysis, with soluble protein contents >27 g per liter and *in vitro* digestibility of 90%. The pH-shift process (ISP) with emulsion breaking techniques is a promising bio-refinery for cold production of gel-forming proteins (and also high-quality fish oil) from salmon byproducts. The process could also be extended after some modifications to herring byproducts (122). In another process, fish waste was subjected to anaerobic fermentation in presence of cow dung. Methane, concentrated liquid mineral and fertilizer were primary products, while CO_2_, solid fertilizer and purified water formed secondary products. Economic viability of the process essentially depended on the yield and market price of methane (123). A case study for industrialization of a bio-refinery examined processes for fish oil extraction, trans-esterification of the oil with ethanol, and omega-3 PUFA recovery. Fish meal and glycerol were the other products of the refinery. Cost-analysis showed that 870 tons wastes of trout processing could generate 160 and 26.6 tons of proteins and ω-3 rich oil by supercritical CO_2_ fractionation. In accordance with the zero-waste concept, all the bio-refinery by-products were valorized. The biofuel obtained from the bio-refinery met the total electricity needs of the plant and provided more than 45% of the thermal energy needs of the trout processing industry. The study demonstrated that the bio-refinery approach increased competitiveness of the fish processing industry (124). The recovered proteins and lipids could be sources of feed and biogas, respectively. The isolated *N*-acetylglucosamine monomers could be used for novel bio-based polymers (120). While designing a biorefinery intended for handling multiple products including energy, it is important to consider the recommendation of the International Energy Agency, Task 42 that upstream protein extraction prior to the conversion of biomass into “energy” and/or co-valorization of protein-rich agro or process residues adds value and improves the business case (40). A Schematic diagram for finfish waste bio-refinery is depicted in Figure 4.

#### Microalgal bio-refinery

**Figure 5:**
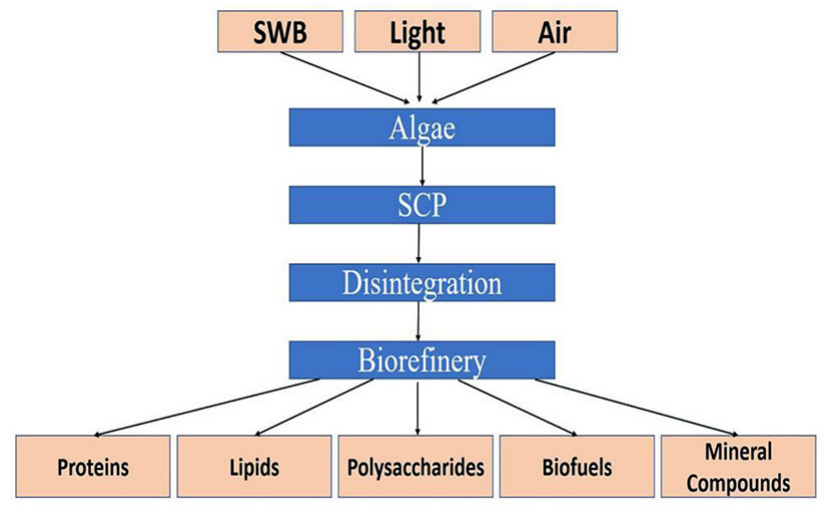
Microalgae-based bioconversion of seafood nutrients into value-added protein and other products. Source: Venugopal (131), with permission from Elsevier.

**Figure 6:**
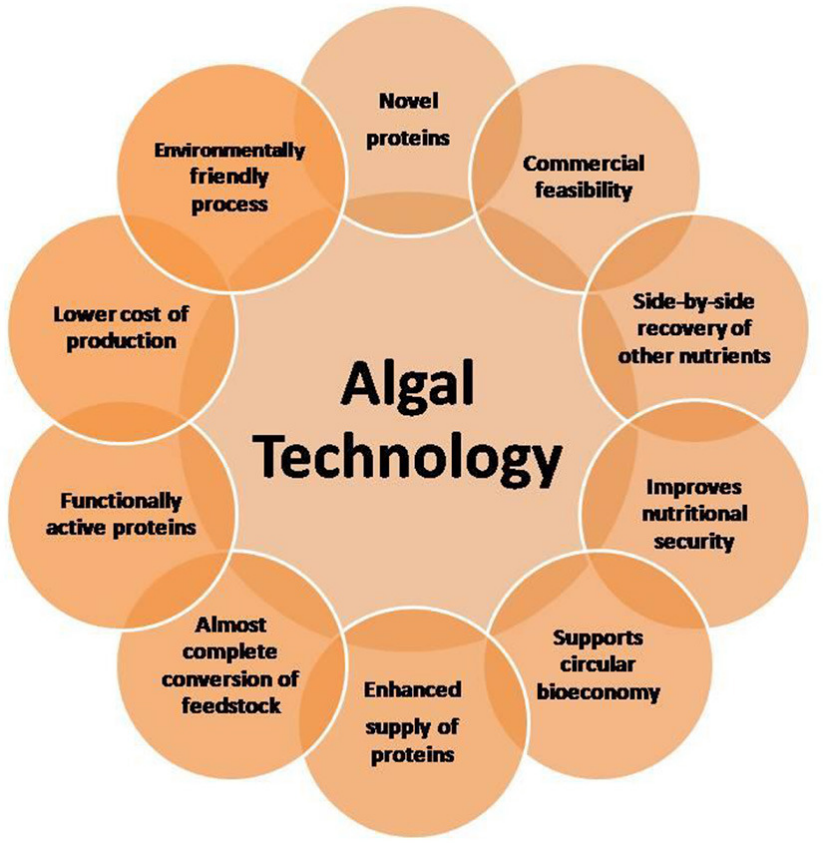
Advantages of algal technology in the valorization of seafood discards and effluents.

Microalgae are increasingly considered major component of multi-products bio-refinery. Algal refinery offers scope for environmentally-friendly and cost-effective seafood waste management (126–128). The combinatorial impacts of utilization of wastewater and CO_2_ by microalgae have led to sustainable and economically feasible biorefineries (76). The SCP produced as a result of algal growth in the seafood media is source of proteins and other ingredients. Recent techniques for the recovery of proteins and other ingredients from SCP make uses of supercritical CO_2_ extraction, enzymatic, microwave-assisted, pressurized-liquid-based extractions, confined impinging jet mixers, and others (129, 130). Figure 5 presents schematic diagram for microalgae-based bioconversion of seafood discards into SCP for onward recovery of proteins and other value-added products. Products derived from SCP are mostly safe to human, animals and plants, and therefore can be used as food additives, nutraceuticals and also for agricultural purposes. Figure 6 summarizes the benefits of algal technology in the recovery of multiple compounds including proteins from seafood discards. Table 3 points out some biorefineries studied for the valorization of seafood discards.

**Table 3:** Recent studies on biorefineries for the recovery of proteins and other products from seafood side streams.

**Bio-refinery**	**Protein type**	**Other products**	**References**
Lactic fermentation	Hydrolyzed marine protein	Astaxanthin, chitin	(70)
Crustacean shell refinery	Proteins	Chitin, lipids, carotenoids, CaCO_3_	(114)
Shrimp refinery, developmental approach		Chitin	(113)
Shell refinery with deproteinization, demineralization	Protein	Chitin	(12, 118)
Sequential enzymatic, acid–alkaline extraction	Proteins from shrimp cephalothorax	Chitin, chitosan, astaxanthin	(119)
Integrated process for shrimp heads	Protein hydrolyzate	Chitin, carotenoids, glycosaminoglycans	(116)
pH-shift process, salmon backbone	Gel forming proteins	Oil	(92)
pH shift process for herring and salmon backbone	Proteins	-	(122)
Fish (trout) waste	Proteins	PUFA, glycerol, liquid biofuel	(124)
Proteolysis of fish waste	Protein hydrolyzate	Food, fertilizer for organic farming	(132)
Proteolysis and fermentation	Gelatin, FPH, peptides, peptones	Oil	(121)
Successive inoculation of shrimp wastes by *L. brevis* and *R. oligosporus*	Protein hydrolyzate	Chitin, astaxanthin	(117)
Integrated refinery for chitin-rich bio-waste	Proteins	Lipids, chitin, chitin monomers	(120)

#### Challenges facing operations of a marine biorefinery

Veríssimo et al. (112) observed that biorefineries are sophisticated multi-step systems, requiring expertise in all stages of manufacturing, in addition to a clear vision of all raw materials, residues, and products. The attributes of a marine biorefinery are low cost of raw material, comparatively simple processes, lower energy consumption, and high productivity (131). However, valorization of fishery products can face challenges. Properties of fishery side streams can vary substantially depending on the types of the product, season, and location of the catch. It is important that fresh feedstock is always preferred to avoid degradation of the biomass. Other challenges for a successful marine bio-refinery include scaling up of the lab scale process, required purity of the isolated compounds, price, and acceptance of the product as well as commercial feasibility of the process. Challenges also arise particularly when the seafood waste can be handled by more than one processing route. Maintaining high productivity is an important requirement at the industrial application level. Stable integrative platforms for sequential or simultaneous recovery of by-products in an efficient manner are fundamental in making seafood biorefineries more economical and sustainable (132). For a successful biorefinery expertise is required in the fields of biotechnology, chemistry, chemical engineering design, algal cultivation technology, predictive environmental sciences and economics and also their sound integration for optimal product recovery. The green techniques must be evaluated for applications at pilot scale before industrial exploitations. Despite rapid advancements in algal biorefineries, the SCP production and extraction processes are in nascent stage. Other technical challenges relate to particle size, pre-treatment, extraction methods, evaluation of bioavailability of nutrients, interaction with other ingredients, nutritional, biotechnological and sensorial aspects, and safety concerns (67). A marine bio-refinery needs to run based on the principles of circular and blue economy model (126). The bottlenecks and challenges facing microalgal bio-refinery and solutions for successful industrial scale operations have been pointed out (127).

## Properties of recovered proteins and their uses

The proteins recovered from seafood side streams are refined composites of myofibrillar and sarcoplasmic proteins that can have interesting nutritional and functional properties. These proteins can be dried and concentrated into a stable fish protein isolate (FPI). Such FPIs normally contains 60 to 70% proteins, which maintain their properties up to 6 months at 5°C, but loses them rapidly at 30°C. Its fat content is a critical issue because when it is oxidized a strong and often rancid flavor is produced. The deterioration can be prevented by eliminating of oxygen from the package (133–135). Incorporation of *Solanum nigrum* extract, a natural immune booster, at 1% level in the product can control lipid oxidation and enhance shelf stability at ambient conditions (136). Alternately, an extract of brown algae *Fucus vesiculosus* can improve the flavor (137). The comparatively mild processes, described earlier, may not adversely affect structural or functional properties of isolated proteins, unlike chemical extraction processes. For example, the proteins recovered from lobster heads were mostly intact (molecular weights 50 to 150 kDa), rich in EAA and had umami taste. The *in vivo* digestibility of these proteins can be in the range of 85 to 98% (15, 16), Cooking, in general, enhances the digestibility of fish proteins in the digestive system. The whole proteins isolated by ISP, algal or other processes are more or less intact, which can be dehydrated to get fish protein isolate (FPI) (89). Proteins extracted in a shell refinery retained their natural state and amino acid contents (115).

Enzymatic or microbial extraction processes lead to fish protein hydrolyzates (FPHs), which consist of peptides of varying sizes. FPH is made by enzymatic digestion of proteins at optimal conditions of pH and temperatures, required by the proteolytic enzymes, which can be from plant (e.g., papain, ficin, etc.) animal (trypsin, pancreatin) or microbial (pronase, alcalase) sources. The hydrolyzed material is decanted and centrifuged to remove scales and bones. The soluble fraction is concentrated, preferably by spray drying. Usually the yield of FPH is about 14% of the raw material. The degree of hydrolysis, which is expressed as the percentage of soluble α-amino nitrogen, is important in determining the functional and bioactive properties of the preparation. By using different fish species, enzymes and optimal digestion conditions, a wide range of FPHs can be prepared (23, 29, 138).

### Nutritional value

The health promoting effects of seafood have chiefly been attributed to nutritive proteins, besides the long-chain n-3 PUFA, particularly, eicosapentaenoic acid (EPA) and docosahexaenoic acid (DHA). The nutritional and health benefits of proteins isolated from side streams are attributed essentially to release of bioactive peptides during their digestion in the body. Therefore, consumption of seafood provides various health benefits (22, 139, 140). The nutritional values of proteins are determined by animal feeding experiments, which include the nitrogen balance method based on protein digestibility, determination of protein efficiency ratio (PER, i.e., weight gained per g of protein consumed), net protein utilization (NPU, i.e., ratio of amino acid converted to protein in the body to the amino acids intake), and biological value, a measure of absorption and utilization of protein by the living organism (141). Enzymatic digestion of proteins in the digestive system gives rise to many peptides and amino acids, which can be absorbed in the intestine. The amino acid score (AAS) of a protein is indicative of its nutritional quality; an AAS score of 100 means high protein quality [(142); SELFNutritionData http://nutritiondata.self.com/, accessed 15 June 2022]. Shellfish proteins have high nutritional value (22). The crude proteins from shellfish discards had amino acid score (AAS), chemical score (CS), and essential amino acid index (EAAI) greater than or close to 1.00, indicating their nutritional values (38). Freeze-dried stick-water of Pollock, cod and salmon contained 70 to 86% proteins. All the samples had more than 95% of digestibility. Their calculated PER values ranged between 1.6 and 1.8 (143). The intake of cod protein provided beneficial effects to diabetic patients by decreasing serum triacylglycerol (TAG), non-esterified fatty acids (FA), and alanine aminotransferase (144). The proteins from southern rock lobster shells had a digestibility comparable to that of the egg protein (15). The isolate from cooked snow crab effluents contained 59% proteins, besides rich contents of minerals, carbohydrates, lipids, and also flavor compounds. The proteins had up to 25% of EAAs, suggesting its nutritive value (98). Bleakley and Hayes (145) observed that the proteins obtained through algae-based processing of seafood discards are on par with soybean and egg proteins in their nutritional values, making them valuable human dietary supplements.

### Functional properties

Functional properties of proteins and other food macromolecules contribute to structural, mechanical and sensory properties of food items. These properties influence behavior of food systems during processing, storage, and consumption. They are determined by the food environment, including presence of water, salt and other compounds, product pH and processing treatments. The important functional properties of proteins are solubility, thermal stability, gelation, emulsifying, foaming, fat binding and water binding abilities (24, 146). The proteins from seafood side streams maintain their functionality because they are extracted under mild conditions. The proteins in surimi powder have good functional properties, such as gelation, water holding capacity, emulsifying and foaming properties (34). Interactions of proteins among themselves and also with other food ingredients, particularly, polysaccharides and lipids have profound influence on food consistency, texture and flavor. These interactions are mainly influenced by the product pH, ionic strength, conformation, charge density and concentration and temperature (147). Kobayashi and Park (148) examined influence of blending of two protein isolates, namely FPI prepared from carp and Alaska surimi. As the proportion of carp FPI increased, surface hydrophobicity and surface reactive sulfhydryl (SRSH) contents increased significantly, indicating that the degree of fish protein unfolding prior to gelation was much higher than surimi alone. The effects of mixing surimi and FPI on gel functionality (hardness, cohesiveness, and whiteness) exhibited a linear pattern when the proportion of surimi was larger than or equal to that of FPI. However, there were no linear relationships when the proportion of FPI exceeded that of surimi. Table 4 shows the functional roles of muscle proteins in a food system.

**Table 4:** Functional roles of muscle proteins in a food system.

**Function**	**Mechanism**	**Food examples**
Solubility	Hydrophilic nature, Entrapment of water through hydrogen bonding	Soup, dispersion
Viscosity	Water binding, hydrodynamic size and shape, thickening	Salad dressings, dessert, gravies, soup
Water holding capacity	Hydrogen bonding, ionic hydration	Meat, sausage, bread, cake
Gelation	Water entrapment, network-formation, matrix formation	Meat, sausage, bread, cake, cheese
Interactions with proteins, polysaccharides and lipids	Hydrophilic, ionic, hydrogen bonding	Meat, sausage, bread, cake, cheese
Elasticity	Hydrophobic bonding, sulfide cross-links	Meat, bakery
Emulsification	Oil adsorption and film formation at interfaces	Meat, sausages, bolognas, soup, cakes
Foaming	Entrapment of air and film formation	Whipped toppings, ice cream, cakes, deserts
Fat flavor bonding	Hydrophobic bonding and entrapment	Low fat bakery products, desserts

Adapted from Damodaran and Paraf (24) and Venugopal (23).

Foaming and emulsifying properties of the proteins were unaffected or slightly improved during their recovery by membrane filtration (43). Proteins derived from lobster heads have high solubility, and emulsification capacities. Their hydrolyzates also possessed excellent emulsifying property (15–17). The peptide-rich hydrolyzates of carp muscle proteins and also the fish collagen possess interesting functional properties (149). Solubility of fish peptides ranges between 50 to 96%, emulsifying activity, 25 to 270 mL per g, foaming capacity 23 to 240%, water holding capacity, 2 to 7 mL per g, and fat binding capacity, 1 to 6 mL per g (150). Fat blocking properties of fish proteins are attributed to their hydrophilic properties (151).

### Bioactivities

The peptides generated during protein digestion have a relatively short length of 2 to 9 amino acids. The peptides are usually resistant to the action of peptidase enzymes in the digestive system. Fish peptides have interesting bioactivities such as antioxidant, antimicrobial, antihypertensive, anti-inflammatory, anti-hyperglycemic, annticoagulant, immuno-modulatory, anticancer, and other activities (1, 152, 153). Such bioactive peptides have also been prepared from proteins recovered from effluents from processing of herring (154), and also from tuna dark muscle protein (155). Low molecular weight peptides (1 to 5 kDa) in general have significant antioxidant activities (156, 157). Depending on their structure and bioactivities, marine peptides have demonstrated palpable effects against diseases such as blood pressure, inflammation, bone degeneration, cancer, diabetics, aging and others. Considerable attention has been devoted to angiotensin converting enzyme (ACE), which makes blood vessels constrict resulting in increased blood pressure. The problem can be addressed by ACE-inhibitory peptides, present in FPH, which help in the prevention of hypertension. These bioactivities make FPHs good dietary supplements (28, 158). Their diverse bioactivities make peptide-rich hydrolyzed seafood proteins including collagen find valuable medicinal applications (25, 150, 152, 153, 156, 158, 159).

### Food and other applications of recovered proteins

In view of their interesting functionality and bioactivities, protein powders from seafood discards such as crustacean and finfish heads are good protein supplements, additives and nutraceuticals. FPIs can be used to improve water holding capacity, oil absorption, gelling activity, foaming capacity and emulsifying properties of food products. Surimi powder offers practical advantages in industrial applications such as easy handling and low distribution costs (34). These proteins can also find uses as texturizers binders, dispersing agents, and emulsifiers in a variety of restructured food products (135, 160). They have been used in restructured products such as crab and lobster meat analogs, fish balls, burgers, fish sausages and also in novel protein bars (16, 89, 148). Animal liver protein and its hydrolysates can have possible applications in food and healthcare (161, 162). FPHs from blue whiting have been used for beverage fortification (163). Proteins from yellow fin tuna roes, extracted by the ISP process, can be additive in noodles, confectionery, baking, and surimi-based products (164). The synergistic interactions of proteins and polysaccharides in their mixed systems could obtain various colloidal structures that can have promising applications in the food industry (147). Bioactive protein hydrolysates and also enzymes extracted by green processing of crab discards can have food applications (86). Hydrolyzed collagen (HC) is a group of peptides (3 to 6 KDa) that is widely used in food, pharmaceutical, cosmetic, biomedical, and leather industries (165). Bakery and pasta products, due to their popularity, offer best scope carriers for marine functional ingredients (166). Similarly, delivering of peptides and other nutraceuticals through dairy products is also highly feasible (167).

Apart from food uses, recovered proteins can have other applications also. Proteins recovered from crab, lobster and other seafood discards or process effluents can also find uses as fertilizers, plant bio-stimulants, and animal feed (5, 17, 20, 86, 89). A reasonable addition of FPH in aqua feeds can improve growth, feed consumption, immune functions and disease resistance of fish (132, 168). The protein isolates can be converted into free-flowing thermo-stable protein dispersion making use of its ability to undergo gelation under mild acidic conditions. Such dispersions can be used as protein coating to extent chilled shelf life of fishery products and for the preparation of fermented sauces (169), Seafood proteins including gelatin can also find uses in protein-based composite biomaterials like nano-components, hydrogels, films, fibers, emulsions and foam, and other materials. Such systems provide mechanical properties, degradability, biocompatibility, and functional properties necessary for carrier systems for drug delivery of nutraceuticals (170, 171). Proteins from Nile tilapia waste were used to prepare biofilms (172). Marine collagen is promising biocompatible alternative to mammalian collagen. Fish collagen and gelatin have applications in pharmaceutical, biomedical, leather, cosmetics, and tissue-engineering industries due to their unique structural and functional features. They can be used for microencapsulation of nutraceuticals, enhancement of sensory properties of low-fat foods (flavor), food emulsifiers, stabilizers, and foaming agents (173–176).

### Economic aspects

The potential availability of proteins from seafood side streams as valuable nutrient can makes significant contributions to fill the supply-demand gap for proteins. Microbial fermentations using GRAS status organisms have commercial advantages (49). Algae-based bioconversion is driven by solar energy and therefore grossly economical to transform seafood nutrients into algal proteins. A successful microalgae based bio-refinery for seafood waste bioconversion can promote zero-waste based circular bio-economy (126, 177, 178). In addition, the recovery of proteins through algal bio-refinery and other ingredients is attractive from the perspective of food security and sustainability and also reduce environmental problems (179). Studies have shown that shell biorefinery can be economically and environmentally viable for the valorization of shell waste at a price of UD$ 0.15 per kg (118). Economic benefits of some marine biorefineries have also been suggested (120, 124). Some economic aspects of production of protein powder have been pointed out. It has been calculated that even 20% of the discards can generate about 8,000 Kt of additional proteins (8). However, currently many green technologies including the emerging non-thermal technologies for the recovery of seafood proteins are still in the developmental stage (44, 45, 180). The technology for recovery of proteins from the SCP needs to be improved. Protein isolates may not be currently available in a commercial scale (148). Industrial scale productions have been suggested to be economically feasible for the recovery of gelatin and other ingredients from processing discards of catfish tuna, and shrimp (16). Success in commercial production of proteins from seafood discards can minimize malnutrition and hunger, particularly prevalent in developing countries (181). The efforts can meet the Waste Framework Directive of EU of 2008, also shared by the US Environmental Protection Agency (182). Furthermore, recovery of nutritive proteins can favor circular bioeconomy, in particular, blue economy, protect the environment and improve seafood sustainability (131). At present there is a great gap between the actual performances of a bio-refinery in relation to expectation in bio-economy (183). Nevertheless, globally, seafood bio-refinery is expected to experience a strong rise. At least 400 manufacturers are currently reported to be involved in this new venture in the European Union (35). It is encouraging to note that global sales of fortified/functional foods reached $292 billion in 2021, up from $274 billion in 2020, per Euromonitor (184). FPH market is likely to grow significantly during 2020 to 2027 (185). The scenario can encourage stakeholders take more interests in green processing of seafood side streams, particularly in the light of rising demand for functional proteins. Successful integration of green chemistry and blue economy principles into ocean-based industries can help a more sustainable, profitable, and conscious ocean economy (112).

## Conclusions

Seafood side streams are good sources of nutritive proteins. In recent times, marine biotechnology has made rapid advancements to recover proteins and other bioactive compounds from seafood discards. A variety of green approaches, as outlined in this article, can recover these proteins. These processes can be appropriately integrated under a bio-refinery approach, to extract not only proteins but also other valuable ingredients from seafood discards. Biotransformation of food waste using microalgae is emerging as green and economical process to recover proteins and also other functionally active compounds. High extraction rates, simpler processes, lower production costs and high productivity are some of the advantages of a successful marine bio-refinery. Further research is necessary to optimize bio-refining of seafood waste for commercial scale protein recovery. New technologies can encourage startup companies focused on global problems in nutrition and health. Close collaboration between fish processing plants and by-product utilization facilities can have significant success in this regard. It is anticipated the information provided in this article will encourage commercial level studies to make use of seafood side streams as source of functional proteins. Efforts on these lines can reduce demand-supply gap of nutritive proteins. Such efforts also improve food security, seafood sustainability and blue economy.

## Author contributions

VV conceived the work, prepared draft, edited, and finalized the article. AS provided additional literature and helped in designing Tables and Figures.

## Funding

We have received 60% waiver of Article Processing Fees.

## Conflict of interest

The authors declare that the research was conducted in the absence of any commercial or financial relationships that could be construed as a potential conflict of interest.

## Publisher's note

All claims expressed in this article are solely those of the authors and do not necessarily represent those of their affiliated organizations, or those of the publisher, the editors and the reviewers. Any product that may be evaluated in this article, or claim that may be made by its manufacturer, is not guaranteed or endorsed by the publisher.
